# *Campylobacter jejuni* Biofilm Control with Lavandin Essential Oils and By-Products

**DOI:** 10.3390/antibiotics11070854

**Published:** 2022-06-25

**Authors:** Dina Ramić, Janja Ogrizek, Franz Bucar, Barbka Jeršek, Miha Jeršek, Sonja Smole Možina

**Affiliations:** 1 Department of Food Science and Technology, Biotechnical Faculty, University of Ljubljana, Jamnikarjeva 101, 1000 Ljubljana, Slovenia; dina.ramic@bf.uni-lj.si (D.R.); ogrizek.janja@gmail.com (J.O.); barbka.jersek@bf.uni-lj.si (B.J.); 2 Department of Pharmacognosy, Institute for Pharmaceutical Sciences, University of Graz, A-8010 Graz, Austria; franz.bucar@uni-graz.at; 3 Slovenian Museum of Natural History, Prešernova Cesta 20, 1001 Ljubljana, Slovenia; mjersek@pms-lj.si

**Keywords:** *Campylobacter jejuni*, biofilm, adhesion, intercellular signaling, lavandin formulations

## Abstract

The food industry is constantly struggling with one of the most prevalent biofilm-forming and food-borne pathogenic bacteria, *Campylobacter jejuni*. Different approaches are used to control biofilms in the food production chain, but none is fully effective. In this study, we aim to produce and determine the chemical profile of essential oils (EOs), ethanolic extracts of flowers prior to distillation (EFs), and ethanolic extracts of post-distillation waste material (EWMs) from *Lavandula × intermedia* ‘Bila’, ‘Budrovka’ St Nicholas and ‘Budrovka’, which were further used to reduce *C. jejuni* intercellular signaling, adhesion, and biofilm formation, as well as to test their antioxidant activity. Glycosides of hydroxycinnamic acids were the major constituents of both types of lavandin ethanolic extract, while linalool, linalyl acetate, 1,8-cineol, and camphor were the major compounds found in lavandin EOs. Tested EOs showed the best antibacterial activity with a minimal inhibitory concentration of 0.25 mg/mL. Lavandin EFs proved more effective in reducing *C. jejuni* intercellular signaling and adhesion compared to lavandin EOs and EWMs, while lavandin EOs showed a slightly better effect against biofilm formation. Interestingly, the best antioxidant activity was determined for lavandin EWMs. A positive and moderate correlation was found between the reduction of *C. jejuni* intercellular signaling and adhesion, as well as between adhesion and biofilm formation. These findings mean novel bacterial targets are of interest for biofilm control with alternative natural agents throughout the whole food production chain.

## 1. Introduction

Microbial biofilms are the predominant form of bacterial lifestyle in industrial environments and protect bacteria from physical trauma, desiccation, and antimicrobial agents [1]. Numerous reports have found that food-borne pathogens persist on food contact surfaces (e.g., plastic, steel, glass, rubber, and wood) in the form of biofilms and affect the quality, quantity, and safety of food products. Moreover, their control is a serious challenge in the food production chain because they cause huge economic and energy losses, damage surfaces and equipment, and lead to continuous contamination of food, posing a major ongoing public health risk [2]. 

Pathogenic bacteria *Campylobacter jejuni* are one of the most common bacterial agents of self-limiting gastrointestinal diseases in humans, but they can also cause more serious neurological disorders, such as Guillain–Barré syndrome [3]. Contaminated surfaces and undercooked poultry meat are the most common vectors of pathogen transmission to humans. Campylobacters represent a severe public health burden in the European Union, where they caused approximately 121.000 intestinal infections in 2020, leading to huge economic losses [4]. A global concern is also the increasing prevalence of antibiotic-resistant and biocide-resistant strains of *C. jejuni*, isolated mainly from poultry [5,6].

Although Campylobacters are considered susceptible bacteria, they survive in environments outside their natural habitat, i.e., intestines. Properties such as intercellular signaling with AI-2 signaling molecule, motility, and chemotaxis allow them to form biofilms or colonize existing biofilms on abiotic surfaces such as polystyrene, glass, and stainless steel. Within biofilms, Campylobacters are protected from antimicrobial agents that penetrate the biofilm matrix slowly and poorly [7]. Various approaches are used to control Campylobacters in the food production chain, but none is fully effective [8]. Therefore, the right approach is needed to control Campylobacters and their biofilm formation in the food production chain at all stages, from primary production to slaughter, processing, and sale of meat. With increasing concerns about antibiotic resistance and environmental impacts, conventional antibiotics, biocides, and preservation methods are being replaced with naturally occurring alternatives that are recognized as safe (GRAS) and have a broad spectrum of antimicrobial activity, including prevention of intercellular signaling, adhesion, and biofilm formation (interrelated bacterial characteristics that are necessary for biofilm establishment) [9,10,11,12].

The plants from the *Lamiaceae* family, genus *Lavandula*, are an inexhaustible source of biologically active phytochemicals with great antimicrobial, anti-biofilm, and antioxidant properties. The genus *Lavandula* includes 39 species, numerous hybrids, and about 400 registered cultivars grown generally in the Mediterranean region [13]. Three *Lavandula* species are used for commercial essential oil (EO) production: *Lavandula angustifolia* Mill. (true lavender), *Lavandula* × *intermedia* Emeric ex Loisel syn. *L. hybrida* L. (lavandin) and *Lavandula latifolia* Medicus (spike lavender) [14]. Nowadays, the cultivation of lavandin, a natural sterile hybrid derived from a cross between *L. angustifolia* and *L. latifolia*, has become increasingly popular because of higher EO yields in comparison to true lavender (120 kg/ha compared to 40 kg/ha). It is preferred for personal care and hygiene products, industrial and household cleaners, as well as for antiseptics, antifungals, and insecticides [15,16]. In this study, flowers of indigenous Croatian cultivars *Lavandula* × *intermedia* ‘Bila’, ‘Budrovka’ St Nicholas (SN), and ‘Budrovka’ were used for the first time to prepare EOs and ethanolic extracts in order to test their anti-biofilm and antioxidant activity, features important for ensuring food safety and quality. 

Higher production of EO consequently resulted in increased accumulation of by-products, i.e., waste materials and hydrolates gained after EO distillation [17,18]. These by-products remain a potentially important source of potent phytochemicals, as shown by the results for spike lavender (which have great antioxidant properties) and lavender hydrolates (which have antifungal and antibacterial properties) [19,20]. The reuse of such natural waste material is environmentally friendly, which makes it more popular than synthetic disinfectants that are often used in the food industry and can lead to additional unnecessary chemicals in the environment [21]. Our previous study has shown that *L. angustifolia* waste material had a promising anti-biofilm effect against pre-established *C. jejuni* biofilms [22], but in this study, lavandin waste materials were used to target *C. jejuni* properties, i.e., intercellular signaling, adhesion, and biofilm formation to prevent *C. jejuni* biofilm establishment. 

The aim of this study was to find potential antimicrobials that will be able to prevent or reduce *C. jejuni* National Collection of Type Culture (NCTC) 11168 biofilm formation on an abiotic surface. For that purpose, dried flowers of *Lavandula* × *intermedia* ‘Bila’, ‘Budrovka’ SN, and ‘Budrovka’ were used to produce EOs, ethanolic extracts of flowers prior to distillation (EFs), and ethanolic extracts of post-distillation waste material (EWMs). Afterward, their chemical characterization was performed. *C. jejuni* intercellular signaling and adhesion were used as targets to prevent or reduce biofilm formation in its early stages. Further, *C. jejuni* biofilm formation, with the addition of lavandin formulations at subinhibitory concentration, was monitored in order to determine how preventive measures affect *C. jejuni* biofilm establishment. A correlation between intercellular signaling, adhesion and biofilm formation was determined. Finally, the antioxidant activity of *L.* × *intermedia* formulations was also explored.

## 2. Results

### 2.1. Chemical Composition of Lavandin Ethanolic Extracts and EOs

The lavandin ethanolic extracts (EFs and EWMs) were analyzed for their phenolic compounds using LC-PDA-ESI-MS analysis. Glycosides of hydroxycinnamic acids were the major constituents of both types of lavandin ethanolic extract. Rosmarinic acid was found in both types of ethanolic extracts; salvianolic A and 3-(3,4-dihydroxyphenyl) lactic acid were only detected in the EWMs. The latter can be easily derived from rosmarinic acid by ester hydrolysis. In addition, flavones apigenin-7-O-glucoside and ladanein were detected. Flavonoids only had a minor role in the composition of the lavandin ethanolic extracts (EFs and EWMs). Table 1 summarizes the peaks that were identified for both types of lavandin ethanolic extract. It is interesting to note that the yield of ethanol extraction was higher for waste materials (EWMs) than for dried flowers (EFs) (Figure S1). Supplementary Figures S2 and S3 illustrate representative UV chromatograms for EFs and EWMs, respectively. To see the difference in composition between both types of extracts, EF and EWM, a comparison of peak areas of individual compounds, i.e., quantification of their relative amounts, is presented in Supplementary Figure S4.

According to the gas chromatography–mass spectrometry (GC–MS) analysis, used lavandin EOs belong to *Lavandula × intermedia* ‘Bila’, L. × intermedia ‘Budrovka’ SN, and L. × intermedia ‘Budrovka’ (Table 2). Linalool was the most represented terpene alcohol in the chemical composition of tested EOs, where lavandin EO ‘Bila’ contained 40.4%, lavandin EO ‘Budrovka’ SN 43.1%, and lavandin EO ‘Budrovka’ 47.2% linalool. Major differences were observed in the content of linalyl acetate, where lavandin EO ‘Bila’ contained 6.6%, lavandin EO ‘Budrovka’ SN 5.3%, and lavandin EO ‘Budrovka’ 26.7% linalyl acetate. Interestingly, lavandin EO ‘Bila’ and lavandin EO ‘Budrovka’ SN were comparable in composition, whereas lavandin EO ‘Budrovka’ showed remarkable differences, aside from its higher linalyl acetate content and its low content of lavandulol, endo-borneol, (Z)-β-ocimene, camphor, terpinene-4-ol and (E)-β-farnesene. In contrast, the content of 1,8-cineol was similar in all EOs (Table 2).

### 2.2. Anti-Campylobacter Activity of Lavandin Formulations

In order to evaluate the anti-Campylobacter activities of the lavandin EOs and ethanolic extracts (EFs and EWMs), their minimal inhibitory concentration (MIC) against *C. jejuni* NCTC 11,168 and *C. jejuni* 11168ΔluxS were determined (Table 3). The lavandin formulations showed antimicrobial efficacy against *C. jejuni* NCTC 11168 at a concentration ranging between 0.25–1 mg/mL. The most favorable effect was shown by samples from the group of EOs: the MICs for all EOs were 0.25 mg/mL. Slightly weaker antimicrobial activity was shown by a group of samples of ethanolic extracts, where lavandin EFs showed better performance compared to lavandin EWMs (Table 3). The same MIC values were also determined against *C. jejuni* 11168ΔluxS, where concentration ranged between 0.25–1 mg/mL. Here, samples of EOs showed the best antimicrobial activity, while lavandin EWM proved to be less effective (Table 3).

Determining the antibacterial activity of the lavandin formulations was crucial for further experiments. Based on the determined MIC values, a subinhibitory concentration (0.25 × MIC) was calculated to avoid the effect of the lavandin formulations on bacterial growth during the experiments (Table 3) [25]. Thus, the focus of this study was to monitor potential changes in *C. jejuni* properties that are important for biofilm establishment, i.e., intercellular signaling, adhesion, and biofilm formation, while exposed to lavandin formulations at subinhibitory concentration.

### 2.3. Modulation of Campylobacter Intercellular Signaling by Lavandin Formulations

The effect of lavandin formulations on the intercellular signaling of *C. jejuni* was verified indirectly by measuring the emitted bioluminescence of the biosensor strain *Vibrio harveyi* MM30. This biosensor was chosen because of its mutation in the luxS gene, which consequently does not synthesize AI-2 signaling molecules but can detect external AI-2 released into the growth medium by *C. jejuni* [26,27]. The intensity of the bioluminescence signal is proportional to the signal concentration in the tested spent medium (SM) [27].

*C. jejuni* NCTC 11168 and *C. jejuni* 11168ΔluxS were cultivated without or with the addition of the lavandin EOs and ethanolic extracts (EFs and EWMs) at a subinhibitory concentration (0.25 × MIC). Before determining the effect of lavandin formulations on the intercellular signaling of *C. jejuni*, the colony-forming units (CFUs) were determined to verify the effect on bacterial growth. Indeed, the used formulations did not significantly affect the growth of *C. jejuni* NCTC 11168 and *C. jejuni* 11168ΔluxS at a concentration of 0.25 × MIC (*p* > 0.05) (Supplementary Table S1). SMs of *C. jejuni* NCTC 11168 and *C. jejuni* 11168ΔluxS were further analyzed with the biosensor *V. harveyi* MM30, and emitted bioluminescence was measured for 15 h. The reduction of bioluminescence was calculated using Equation (1). This was the indirect measure for the reduction of *C. jejuni* intercellular signaling.

**Figure 1:**
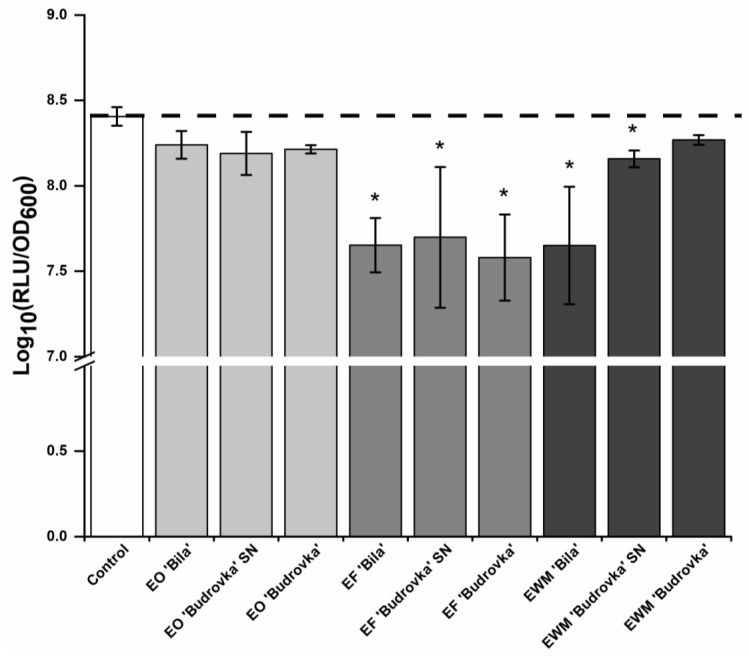
Effect of different lavandin formulations (EOs, EFs and EWMs) at subinhibitory concentration (0.25 × MIC) on the intercellular signaling of *C. jejuni* NCTC 11168. *C. jejuni* NCTC 11168 was cultivated for 24 h without or with the addition of lavandin formulations at subinhibitory concentration. Afterwards, SMs were prepared and added to *V. harveyi* MM30 biosensor strain. *V. harveyi* MM30 bioluminescence response was the indirect measure for *C. jejuni* intercellular signaling. Log_10_ average values ± SD are shown. * *p* < 0.05, vs. control. (EO, essential oil; EF, ethanolic extract prior to distillation; EWM, ethanolic extract of post-distillation waste material).

All lavandin EFs significantly reduced *C. jejuni* intercellular signaling (*p* < 0.05) (Figure 1). Lavandin EWM ‘Bila’ and EWM ‘Budrovka’ SN were also successful in reducing *C. jejuni* intercellular signaling (*p* < 0.05), while lavandin EOs did not have a significant effect on *C. jejuni* intercellular signaling (*p* > 0.05) (Figure 1). Lavandin EFs ‘Bila’ and ‘Budrovka’ and lavandin EWM ‘Bila’ reduced *C. jejuni* intercellular signaling by approximately 95% (Supplementary Table S2).

### 2.4. Modulation of Campylobacter Adhesion by Lavandin Formulations

**Figure 2:**
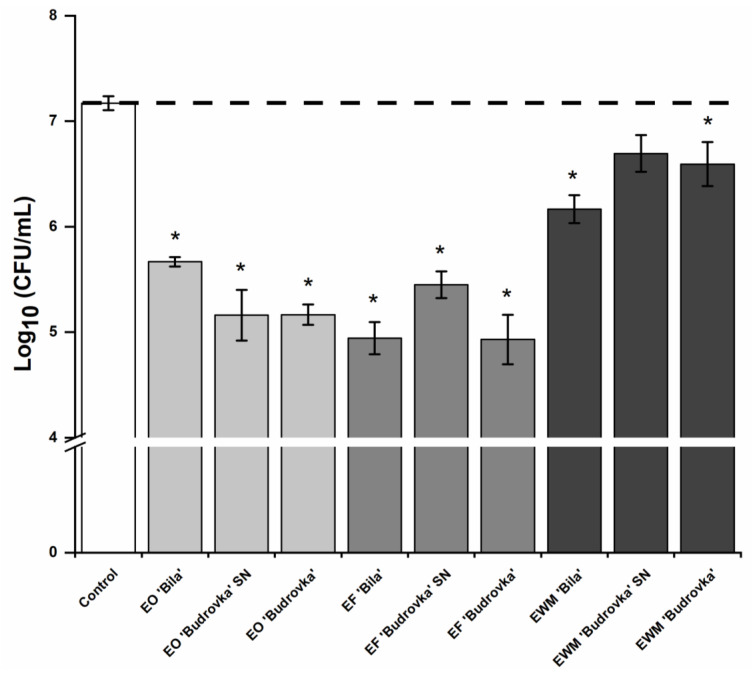
Effect of different lavandin formulations (EOs, EFs and EWMs) at subinhibitory concentration (0.25 × MIC) on the adhesion of *C. jejuni* NCTC 11168 to a polystyrene surface. *C. jejuni* NCTC 11168 was cultivated in a polystyrene microtiter plate without or with the addition of lavandin formulations at subinhibitory concentration in a micro-aerobic atmosphere at 42 °C for 24 h. Attached cells were suspended by sonication and their concentration was determined by plate counting. Log_10_ average values ± SD are shown. * *p* < 0.05, vs. control. (EO, essential oil; EF, ethanolic extract prior to distillation; EWM, ethanolic extract of post-distillation waste material).

Lavandin EOs, EFs, and EWMs were used at a subinhibitory concentration (0.25 × MIC) in order to prevent the adhesion of *C. jejuni* to a polystyrene surface. Results are shown in Figure 2. The adhesion of *C. jejuni* to a polystyrene surface was significantly reduced by all lavandin EOs and ethanolic extracts (EFs and EWMs), with the exception of lavandin EWM ‘Budrovka’ SN (*p* < 0.05). Within the exact group of lavandin formulations, EOs, EFs and EWMs had a similar effect on *C. jejuni* adhesion (Figure 2). 

Lavandin EF ‘Bila’ and ‘Budrovka’ proved slightly more effective, with an almost 99% reduction of adhesion (i.e., a reduction of 2 log_10_). Lavandin EF ‘Bila’ was more effective on *C. jejuni* adhesion compared to EO and EWM ‘Bila’ (*p* < 0.05). There were no significant differences in the effects of EO and EWM ‘Bila’ (*p* > 0.05). Lavandin EO ‘Budrovka’ SN and ‘Budrovka’ were more effective than the lavandin EWMs (*p* < 0.05), but there were significant differences compared to EFs ‘Budrovka’ SN and ‘Budrovka’ (*p* > 0.05). All lavandin EFs were more effective in reducing *C. jejuni* adhesion to the polystyrene surface compared to the lavandin EWMs (*p* < 0.05).

### 2.5. Modulation of Campylobacter Biofilm Formation by Lavandin Formulations

The biofilm formation of *C. jejuni* was observed on a glass surface, which presented the model of an abiotic surface. Lavandin formulations at subinhibitory concentration were used in order to prevent biofilm formation. Coverage of the glass surface with *C. jejuni* NCTC 11,168 biofilm at air/liquid interface was determined after 72 h of exposure to lavandin formulations. Every 24 h, the SM was replaced with a fresh one, where lavandin formulations at subinhibitory concentration were added. This step was important because, in this way, *C. jejuni* was constantly exposed to the same concentration and form of lavandin formulations. 

**Figure 3:**
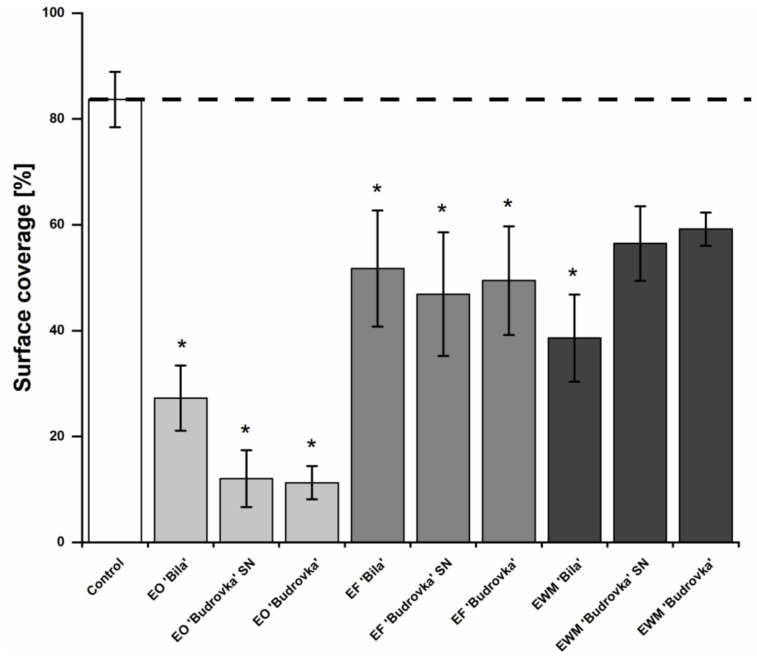
Effect of different lavandin formulations (EOs, EFs and EWMs) at subinhibitory concentration (0.25 × MIC) on biofilm formation of *C. jejuni* NCTC 11168. *C. jejuni* NCTC 11,168 was cultivated on a glass surface without or with the addition of lavandin formulations at subinhibitory concentration in a micro-aerobic atmosphere at 42 °C for 72 h. Every 24 h, the SM was replaced with a fresh Muller–Hinton (MH) broth, where lavandin formulations at subinhibitory concentration were added. After 72 h of incubation, microscopic slides were rinsed and stained with 1% [*v*/*v*] crystal violet. Biofilms were examined at air/liquid interface and surface coverage was measured. Average values ± SD are shown. * *p* < 0.05, vs. control. (EO, essential oil; EF, ethanolic extract prior to distillation; EWM, ethanolic extract of post-distillation waste material).

All lavandin EOs and ethanolic extracts (EFs) significantly reduced the biofilm formation of *C. jejuni* on the glass surface (*p* < 0.05) (Figure 3). Among the lavandin EWMs, only lavandin EWM ‘Bila’ significantly reduced biofilm formation (*p* < 0.05). Once again, it is obvious that, within the exact group of lavandin formulations, EOs, EFs and EWMs had a similar effect on *C. jejuni* biofilm formation. 

Lavandin EOs proved most effective against *C. jejuni* biofilm formation, where lavandin EO ‘Budrovka’ SN and ‘Budrovka’ reduced the biofilm formation by approximately 85%. Interestingly, lavandin EO ‘Bila’ reduced biofilm formation by approximately 67%, and its effect was not significantly different from the effect of lavandin EF ‘Bila’ and EWM ‘Bila’ (*p* > 0.05).

Lavandin ethanolic extracts (EFs and EWMs) had a weaker effect on *C. jejuni* biofilm formation. Among the ethanolic extracts, the most effective was lavandin EWM ‘Bila’, with a biofilm reduction of approximately 44%. The effect of lavandin EWM ‘Bila’ was not significantly different from the effect of lavandin EF ‘Bila’.

### 2.6. Correlation between MIC and C. jejuni Intercellular Signaling, Adhesion and Biofilm Formation

**Figure 4:**
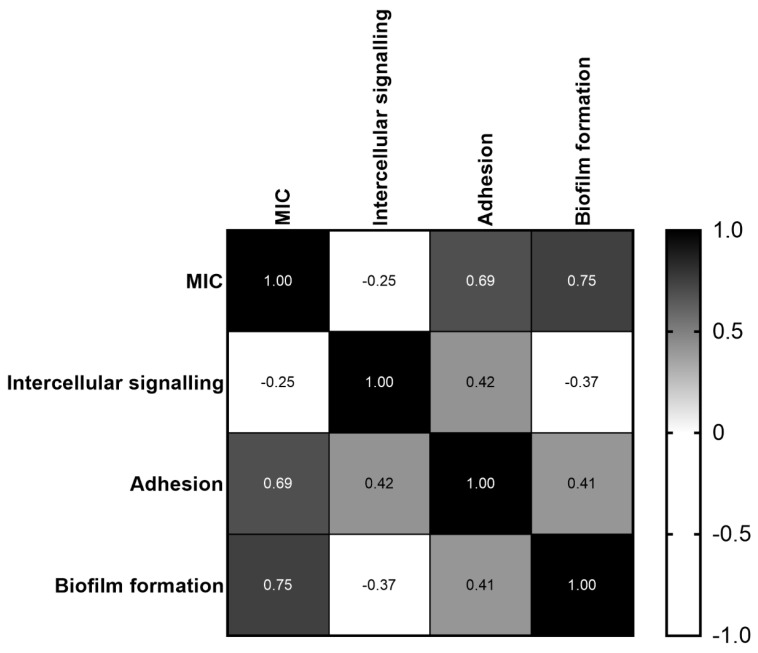
Correlation between MIC of lavandin formulations, *C. jejuni* intercellular signaling, adhesion and biofilm formation. Pearson’s correlation test was used in order to determine the correlation among variables. For the Pearson correlation, an absolute value of 1 indicates a perfect linear relationship. A correlation close to 0 indicates no linear relationship between the variables. The sign of the coefficient indicates the direction of the relationship. Correlation was significant at the level < 0.01.

In order to test whether the measured parameter MIC and *C. jejuni* properties—i.e., intercellular signaling, adhesion, and biofilm formation—were in correlation, Pearson’s correlation test was used (Figure 4). There was a weak and negative correlation (*p* < 0.01) between MIC and *C. jejuni* intercellular signaling (Figure 4), meaning that the increment of MIC leads to the reduction of intercellular signaling. A medium and positive correlation (*p* < 0.01) was found between *C. jejuni* intercellular signaling and adhesion (Figure 4), meaning that the reduction of intercellular signaling leads to the reduction of adhesion. A medium and positive correlation (*p* < 0.01) was also observed for *C. jejuni* adhesion and biofilm formation (Figure 4), meaning that the reduction of adhesion leads to the reduction of biofilm formation.

### 2.7. Antioxidant Activity of Lavandin Formulations

The antioxidant activity of the lavandin formulations was determined using the DPPH radical scavenging assay. Lavandin EOs were tested at a concentration of 40 mg/mL. Lavandin EO ‘Bila’ and ‘Budrovka’ showed similar results, with lavandin EO ‘Bila’ having a scavenging activity of 75.03 ± 11.33% and lavandin EO ‘Budrovka’ having a scavenging activity of 70.85 ± 2.80%. Lavandin EO ‘Budrovka’ SN had better antioxidant activity, with 90.17% ± 0.45% scavenging activity. 

The lavandin ethanolic extracts (EFs and EWMs) were tested for antioxidant activity at a much lower concentration compared to the lavandin EOs. EFs were tested at a concentration of 2.5 mg/mL, while EWMs were tested at a concentration of 1 mg/mL. EF ‘Bila’ and ‘Budrovka’ SN had similar results, with EF ‘Bila’ having a scavenging activity of 86.73 ± 2.81% and EF ‘Budrovka’ SN of 83.54 ± 4.58%. EF ‘Budrovka’ had the best antioxidant activity among the lavandin EFs, with a scavenging activity of 93.87 ± 0.67%. 

All three EWMs had a similar scavenging activity, with lavandin EWM ‘Bila’ having a scavenging activity of 92.16 ± 1.73, EWM ‘Budrovka’ SN of 94.38 ± 1.19, and EWM ‘Budrovka’ of 92.94 ± 0.38%.

## 3. Discussion

Current measures that are used to combat the persistence of *C. jejuni* in the food production chain are not fully effective, so there is a need for new approaches to control *C. jejuni* across the whole food production chain from farm to fork. In order to find potential antimicrobials that will be able to prevent or reduce *C. jejuni* biofilm establishment on abiotic surfaces, dried flowers of *Lavandula × intermedia* ‘Bila’, ‘Budrovka SN, and ‘Budrovka’ were used to prepare EOs, ethanolic extracts of lavandin flowers prior to distillation (EFs) and ethanolic extracts of lavandin post-distillation waste material (EWMs). Prepared lavandin formulations were used to target *C. jejuni* intercellular signaling, adhesion, and biofilm formation in order to combat biofilm establishment. Subinhibitory concentration was used to avoid effects on *C. jejuni* growth.

Prior to the experiments at the biological level, the chemical composition of lavandin EOs, EFs and EWMs was investigated to gain insight into the chemical profile of prepared lavandin formulations. EFs and EWMs had a similar chemical composition, where phenols were the major compounds detected. However, it is important to note that the extraction yield was higher for waste material compared to flowers prior to the distillation (Supplementary Figure S1). This can be due to better accessibility of waste material to the solvent, as by hydrodistillation, the plant material was cooked for the time of hydrodistillation. Therefore, the plant cellular matrix was better solubilized, and extractive compounds were more easily available. Moreover, 3-(3,4-dihydroxyphenyl)lactic acid, the hydrolysis product of rosmarinic acid, as well as salvianolic acid A, could only be detected in the extract from waste materials after hydrodistillation, indicating that artifact formation during hydrodistillation has to be taken into account. In addition, the flavones apigenin-7-O-glucoside and ladanein were detected. A comparable composition was reported for *L. × intermedia* waste material, where chlorogenic acid and a number of flavone glycosides were also found [18]. A similar composition for *Lavandula* ethanolic extracts was also reported in our previous study [22]. Mass spectrometry and UV-VIS data were used for the identification of phenols, so the sugar moieties were only designated as hexosides; however, according to other reports for *Lavandula* spp., glucosidation is most likely to occur [18,28]. 

The complete chemical profile was determined for all three lavandin EOs. Linalool, linalyl acetate, 1,8-cineol, and camphor were the major compounds found in tested EOs. This confirms that tested EOs belonged to the genus *Lavandula*, more specifically to the hybrid *L. × intermedia* [29,30]. The EO of *L. × intermedia* ‘Budrovka’ contained the highest percentage of linalool (47.2%) and linlyl acetate (26.7%) compared to the percentage of linalool and linalyl acetate found in *L. × intermedia* ‘Bila’ and ‘Budrovka’ SN (40.4–43.1% and 5.3–6.6%, respectively). This is especially interesting because *L. × intermedia* ‘Budrovka’ and ‘Budrovka’ SN were cultivated at the same geographic location but in different fields, which indicates that ontogenetic and morphogenetic factors can also influence the chemical variability, either to a quantitative or qualitative extent [31]. 

Among tested lavandin formulations, all three lavandin EOs were shown to possess the best antibacterial activity, with MIC values of 0.25 mg/mL. The antibacterial activity of *L. × intermedia* ‘Budrovka’ and *L. angustifolia* EOs were observed against different Gram-positive and Gram-negative bacteria [29]. In our previous study [22], the same MIC was determined for *L. angustifolia* EO against *C. jejuni*. Regarding the information provided, *C. jejuni* seems most sensitive to *Lavandula* formulations among all tested bacteria. This could be due to the type of microorganism, the inoculum volumes, and the culture medium used, together with the pH, temperature, and incubation time. Type and storage, as well as the method for plant formulation preparation, can also influence antimicrobial activity [32].

EFs and EWMs had a moderate but comparable effect against *C. jejuni*, although weaker than EOs. It is most likely that this can be explained by the glycosidic nature of the major constituents of the ethanolic extracts resulting in decreased cell wall permeability. Nevertheless, EFs and EWMs still contain a diverse pool of bioactive compounds and are effective antibacterial agents. This is in agreement with previous studies on post-distillation thyme waste, pinot noir grape skins and seeds, juniper fruit waste, and *Lavandula* waste material, which also showed great antimicrobial activity [22,33,34].

In further experiments, lavandin formulations were used at a subinhibitory concentration as a novel approach to control biofilm development, focusing on *C. jejuni* properties: intercellular signaling and adhesion. All lavandin EFs and lavandin EWMs ‘Bila’ and ‘Budrovka’ SN significantly reduced *C. jejuni* intercellular signaling, proving to be more effective than the lavandin EOs. It is hypothesized that the signaling molecules were bound to the solid aggregates of the precipitate in the samples of the ethanolic extracts, which became apparent during the preparation of the SMs and were removed along with the bacteria during filtration. Moreover, the process of intercellular signaling can be disrupted by different mechanisms: reducing the activity of receptor protein or synthase; inhibiting the production of signaling molecules; degrading the signaling molecules; mimicking the signaling molecules primarily by using the analogs of signal molecules (e.g., secondary metabolites of natural formulations) [35]. A comparable result was reported for *L. hybrida* EO at subinhibitory concentration, which inhibited the intercellular signaling of *C. jejuni* by approximately 66% [25]. Similarly, a strong effect on the inhibition of *C. jejuni* intercellular signaling was also found for the ethanolic fruit extract *Euodia ruticarpa*, which showed a reduction of more than 90% [36].

Adhesion is a bacterial feature affected by intercellular signaling. It is crucial for the development of *C. jejuni* biofilm, so it is necessary to inhibit it in order to prevent biofilm establishment [9]. A statistically significant effect on the reduction of *C. jejuni* adhesion to a polystyrene surface was confirmed for all tested lavandin preparations, with the exception of the lavandin EWM ‘Budrovka’ SN. There were no statistically significant differences between the anti-adhesion effect of lavandin EOs and EFs. Moreover, a slightly better effect could be observed for lavandin EF ‘Bila’ and ‘Budrovka’ compared to their EOs, where lavandin EF ‘Budrovka’ reduced adhesion by >99%. This is comparable with the results gained for thyme ethanolic extracts [33]. An excellent anti-adhesion effect was also observed for the *L. hybrida* EO, which reduced the adhesion of *C. jejuni* to the polystyrene surface by 96% [25]. This is comparable to the observed results for the lavandin EOs. Similar results for lavender formulations were shown in our previous study [22], where it was confirmed that lavender EOs were able to affect the expression of genes carrying the transcript for the outer membrane proteins involved in the initial adhesion of *C. jejuni* to contact surfaces. Moreover, a moderate and positive correlation was found between intercellular signaling and adhesion, indicating that a decrease in intercellular signaling leads to a decrease in the adhesion of bacterial cells to abiotic surfaces. These results are supported by the research by [25], who also found a correlation between the reduction of intercellular signaling and the reduction of adhesion. Altogether, these findings confirm that formulations from the genus *Lavandula* have great anti-adhesion potential against *C. jejuni* and that intercellular signaling is an important target of *Lavandula* preparations to combat the adhesion of *C. jejuni.*

It is clear that the lavandin formulations successfully reduced *C. jejuni* adhesion, but the most important question was whether the lavandin formulations could reduce biofilm development even after 72 h of incubation. Indeed, all lavandin EOs and EFs, as well as lavandin EWM ‘Bila’, significantly reduced the biofilm development of *C. jejuni* on a glass surface even after 72 h of incubation (*p* < 0.05). A moderate and positive correlation was found between reducing adhesion and biofilm formation, confirming that reducing adhesion is a crucial step in combating bacterial biofilm, but a correlation between reducing intercellular signaling and biofilm was not found, which was expected, as biofilm formation is a multifactorial event and does not only depend on intercellular signaling. If we consider all the facts together, it is evident that intercellular signaling is the primary mechanism that needs to be reduced in order to reduce adhesion and, consequently, biofilm establishment. The lavandin EOs had the most favorable inhibitory effect against biofilm establishment. The latter can be attributed to the higher content of bioactive secondary metabolites in EO. It is interesting that there were no significant differences between the effects of lavandin EO, EF, and EWM ‘Bila’, indicating that waste material can match EO in its effects. Studies have also shown the anti-biofilm activity of linalool against different bacteria [9,29], but not as good as for EO. Naturally, linalool is one of the major compounds found in the tested EOs, but it is important to emphasize that the action of EO comes from the action of all the bioactive compounds found in EO [22]. 

Finally, the antioxidant activity of prepared lavandin formulations was tested. The antioxidant activity of natural compounds is important because it can reduce the oxidation of food products that come to consumers, thus improving food quality [37]. Among all the lavandin formulations, lavandin EWM had the best antioxidant activity and scavenging activity of more than 90% at a concentration of 1 mg/mL. In order to gain a similar effect with lavandin EFs and EOs, a concentration 2.5 times or 40 times higher had to be used. Similar results were found for the ethanolic extract *Ocimum basilicum*, which had better antioxidant activity than the EO of *O. basilicum* [38]. The better antioxidant activity of lavandin ethanolic extract formulations can be attributed to their strongly different chemical composition compared to EOs. For example, ladanein, which was found in the tested ethanolic extracts, is known to be a good antioxidant agent [39]. Moreover, lavandin EWMs had a relatively higher concentration of some identified compounds than EFs (concluded from the mass spectrometry and UV-VIS data), which can explain their better antioxidant activity. Similar results were found for lavandin ‘Budrovka’ EO, which had an IC_50_ value of 21.6 mg/mL [29]. By comparing our results with the research carried out on lavandin ‘Sumiens’, ‘Super A’ and ‘Grosso’, it was recognized that tested cultivars ‘Bila’, ‘Budrovka’ SN, and ‘Budrovka’ had an antioxidant activity that was twice as good [40]. Such an effect is probably the result of synergistic interactions between EOs constituents, as linalool and linlyl acetate had much higher IC_50_ values (218.6 mg/mL or 157.1 mg/mL, respectively) than lavandin EO [29].

## 4. Material and Methods

### 4.1. Chemicals

The MH agar was from BioMéroeux (Marcy-l’Étoile, France), the MH broth was from Oxoid (Hampshire, UK) and the Karmali agar was from Biolife (Milan, Italy). The glycerol solution was from Kemika (Zagreb, Croatia), the phosphate-buffered saline (PBS) was from Oxoid, and the kanamycin, dimethylsulphoxide (DMSO), resazurin, menadione, and Folin–Ciocalteu reagent were from Sigma Aldrich (Steinheim, Germany). The sodium chloride, magnesium sulfate heptahydrate, L-arginine, and 96% ethanol were from Merck (Darmstadt, Germany). The casamino acid was from Thermo Fisher Scientific (Carlsbad, CA, USA).

### 4.2. Lavandin Formulations

Three *Lavandula × intermedia* cultivars (‘Bila’, ‘Budrovka’ SN and ‘Budrovka’) were used in this study. *Lavandula × intermedia* ‘Bila’ was cultivated in Spodnje Pitve, Hvar, Croatia (43°09′06″ N, 16°40′35″ E), while *L. × intermedia* ‘Budrovka’ SN and *L. × intermedia* ‘Budrovka’ were cultivated in Jelsa, Hvar, Croatia (43°09′23″ N, 16°41′04″ E). The samples were collected in the afternoon hours during July 2019. Dried flowers were used to prepare the lavandin EOs and ethanolic extracts (EFs). 

The EOs were prepared by hydrodistillation [41], with about 200 g of flowers distilled in two liters of water in a Clevenger-type apparatus for three to four hours and then stored at 4 °C. The waste material obtained after the hydrodistillation of the lavandin flowers was also used for the preparation of the ethanolic extracts (EWMs).

The ethanolic extracts from the lavandin dried flowers (EFs) and waste material (EWMs) gained after the hydrodistillation process were prepared by a four-to-six-hour ethanol extraction (Soxhlet extraction) of 20 g dried flowers in 150 mL 96% ethanol. These were then concentrated in a rotary evaporator (Laborota 4000; Heidolph Instruments, Germany) at 40 °C and 175 mbar pressure and stored at 4 °C.

### 4.3. Phytochemical Analysis of Lavandin Ethanolic Extracts

The identification of the phenolic compounds in lavandin ethanolic extracts (EFs and EWMs) was carried out using liquid chromatography—photo diode array—electrospray ionization mass spectrometry (LC-MS) following the protocol described in [22] (for details, see Supplementary Methods, LC-MS Conditions). The compounds eluted were determined by their UV-VIS and mass spectra, in comparison with the literature [18,42,43,44,45,46]. 

### 4.4. GC–MS Analysis of Lavandin EOs

The identification of the main compounds in lavandin EOs was carried out by GC–MS following the protocol described in [19] (for details, see Supplementary Methods, GC–MS Conditions). The compounds were identified by their retention indices according to [44] and by comparing their mass spectra with spectral data libraries [23,24,47] and with the laboratory’s own database.

### 4.5. Bacterial Strains and Growth Conditions

*C. jejuni* NCTC 11168 and *C. jejuni* 11168ΔluxS [48] were used in this study. The strains were stored at −80 °C in a 20% glycerol and 80% MH broth. Prior to the experiments, *C. jejuni* NCTC 11168 was subcultivated on Karmali agar, and *C. jejuni* 11168ΔluxS on an MH agar supplemented with 30 mg/L kanamycin for 24 h at 42 °C under micro-aerobic conditions (85% N_2_, 5% O_2_, 10% CO_2_). The strains were further subcultured in an MH broth under the same conditions, and bacterial OD was determined by spectrophotometric measurements of absorbance at 600 nm after the incubation. The inoculum was prepared in an MH broth at 10^5^ CFU/mL for determination of the MICs and assays that targeted *C. jejuni* intercellular signaling and adhesion. For counting, strains were plated on an MH agar under conditions described above in this section, and the colonies were counted and expressed as CFU/mL.

For the autoinducer-2 bioassay, the biosensor strain *V. harveyi* MM30 [22] was used. The strain was stored at −80 °C in a 20% glycerol and 80% autoinducer bioassay (AB) medium composed of NaCl [0.02 g/L], MgSO4 + 7 H2O [0.01 g/L], casamino acid [0.002 g/L], PBS [1 M], L-arginine [0.1 M] and glycerol [50% (*v*/*v*)] [49]. Prior to the experiments, strains were subcultured for 16 h aerobically at 30 °C in an AB liquid medium.

### 4.6. Antimicrobial Potential of Lavandin Formulations

The MICs against *C. jejuni* NCTC 11168 and *C. jejuni* 11168Δ*luxS* were determined by the broth microdilution method, as previously described [50]. Stock solutions of lavandin EOs and ethanolic extracts (EFs and EWMs) were prepared in DMSO at 40 mg/mL. Serial dilutions of stock solutions were performed in an MH broth in a 96-well microtiter plate (NUNC 266 120 polystyrene plates; Nunc, Denmark), after which bacterial inoculum was added, prepared as described in Section 2.5. During experiments, the DMSO in the MH broth did not exceed a concentration of 1% [*v*/*v*], which did not influence the growth of bacteria [25]. In further experiments, a concentration of 0.25 × MIC was used, as this concentration was the first concentration that did not influence the growth of *C. jejuni* [25]. 

### 4.7. Targeting Intercellular Signaling of C. jejuni

Overnight cultures of *C. jejuni* NCTC 11168 and *C. jejuni* 11168ΔluxS were inoculated in an MH broth until OD600 0.1 (10^7^ CFU/mL). For further experiments, the cultures were diluted 100-fold in an MH broth, and an MH broth supplemented with lavandin formulations (EOs and ethanolic extracts [EFs and EWMs]) at subinhibitory concentration (0.25 × MIC). The cultures were further incubated for 24 h at 42 °C under micro-aerobic conditions. After the incubation, bacterial growth was determined using the plate counting method as described previously (Section 2.5), and cultures were further filtrated through 0.2 µm syringe filters to gain an SM where no bacteria were present. SMs were stored at −80 °C until further experiments.

*V. harveyi* MM30 was used for the biosensor assay in order to test the effect of lavandin formulations on *C. jejuni* intercellular signaling. An overnight culture of *V. harveyi* MM30 was diluted 5000-fold in an AB liquid medium to contain approximately 10^3^ CFU/mL and was used in further experiment. *C. jejuni* NCTC 11168 SM (CJ–SM) and *C. jejuni* 11168ΔluxS SM (LUXS–SM), untreated or treated with lavandin formulations at subinhibitory concentration, were added to the suspension of the biosensor to a final concentration of 5% (*v*/*v*) of each (i.e., 10 µL of CJ–SM, 10 µL of LUXS–SM and 180 µL of the biosensor strain) in 96-well white microtiter plates with a transparent bottom (Greiner Bio-One, Kremsmünster, Austria). LUXS–SM, untreated or treated with lavandin formulations at subinhibitory concentration, was used as the blank [5% (*v*/*v*) LUXS–SM, 95% (*v*/*v*) AB medium], while the negative control was a 5% (*v*/*v*) LUXS–SM and 95% (*v*/*v*) *V. harveyi* suspension. The relative luminescence signals, expressed as relative luminescence units (RLU), and a growth of *V. harveyi* MM30, expressed as OD600, after the addition of CJ–SM (treated or untreated), LUXS–SM (treated or untreated), and MH broth, were measured with a microplate reader (Varioskan Lux, Thermo Scientific, Waltham, MA, USA) at 30 min intervals over 15 h at 30 °C. 

The measured values (RLU and OD600) of LUXS–SM (treated or untreated), used as blank, were deducted from the values (RLU and OD600) gained for the bioluminescence response and the growth of the *V. harveyi* MM30 biosensor after the addition of CJ–SM and LUXS–SM (both treated or untreated). Results from the bioluminescence response were normalized with OD600. The reduction of bioluminescence was calculated by Equation (1):

(1) $$\%\;bioluminescence\;reduction=100-\left(\;\frac{11168T-luxST}{11168C-luxSC}\right)\times 100\%,$$

where 11168T is the normalized bioluminescence response of *V. harveyi* MM30 after the addition of CJ–SM treated with lavandin formulations at subinhibitory concentration, luxST is the normalized bioluminescence response of *V. harveyi* MM30 after the addition of LUXS–SM treated with lavandin formulations at subinhibitory concentration, 11168C is the normalized bioluminescence response of *V. harveyi* MM30 after the addition of untreated CJ–SM and luxSC is the normalized bioluminescence response of *V. harveyi* MM30 after the addition of untreated LUXS–SM.

### 4.8. Anti-Adhesion Potential of Lavandin Formulations

The adhesion of *C. jejuni* NCTC 11168 was investigated under treatments with lavandin formulations at subinhibitory concentration. Inoculum was prepared as described in Section 2.5 and treated with lavandin EOs, and ethanolic extracts (EFs and EWMs) at 0.25 × MIC Polystyrene microtiter plates with 96 wells (Nunc 266 120 polystyrene plates; Nunc, Denmark) were prepared as described in [22] and were incubated for 24 h. The adhesion of cells was determined as CFU/mL, as previously described [22,33]. The untreated culture was used as a negative control. 

### 4.9. Anti-Biofilm Potential of Lavandin Formulations

The anti-biofilm potential of lavandin EOs and ethanolic extracts (EFs and EWMs) was evaluated according to the previously reported method [22]. Briefly, in a 50 mL centrifuge tube (Sarstedt, Nümbrecht, Germany), 20 mL of MH broth supplemented with lavandin formulations at subinhibitory concentration (0.25 × MIC) were added and were inoculated with 5% [*v*/*v*] inoculum of *C. jejuni* overnight culture (10^8^ CFU/mL). Autoclaved microscope slides (26 × 76 mm; Deltalab, Barcelona, Spain) were used as a model for a glass surface and were vertically inserted into the centrifuge tube after inoculation of the medium. The cultures were incubated without shaking in a micro-aerobic atmosphere for 72 h at 42 °C, in a damp environment. After 24 h, microscopic slides were transferred to new centrifuge tube where 20 mL of fresh MH broth, supplemented with lavandin formulations at subinhibitory concentration (0.25 × MIC), were added. The same was repeated after an additional 24 h of incubation. Untreated cultures were used as negative controls. After 72 h of incubation, the microscopic slides were rinsed three times with PBS to remove weakly adhered cells and stained with a 1% [*v*/*v*] crystal violet stain. The biofilms on the air/liquid interface were investigated with a light microscope (DM750, Leica, Germany) equipped with a camera (ICC50 W, Leica, Germany) under a 400× *g* magnification. For each sample, five technical replicates of vertically connected images of biofilm at the air/liquid interface were captured (5 × 1 mosaic images; total analyzed surface per image, 1600 μm × 1200 μm). The images were processed using the Fiji program [51] as described in [22], with minor modifications. Based on the image processing, the surface coverage in percent was determined. 

### 4.10. Free Radical Scavenging Activity Assay (DPPH Assay)

The free radical scavenging activities of the lavandin EOs and ethanolic extracts (EFs and EWMs) were evaluated using the stable DPPH radicals as previously described [29]. Briefly, the DPPH was prepared at a concentration of 0.2 mg/mL in 96% ethanol. The lavandin EOs were assayed at a concentration of 40 mg/mL in 96% ethanol, lavandin ethanolic extracts (EFs) at a concentration of 2.5 mg/mL in 96% ethanol and lavandin ethanol extracts (EWMs) at a concentration of 1 mg/mL in 96% ethanol. We added 20 µL of DPPH to 60 µL of the lavandin samples (EOs, EFs, and EWMs) in a non-sterile 96-well polystyrene microtiter plate (Brand, Wertheim, Germany). For blank, 20 µL of 96% ethanol was added to 60 µL of the lavandin samples (EOs, EFs, EWMs), and for a negative control, 20 µL of DPPH solution was added to 60 µL of the 96% ethanol. The microtiter plate was shaken for 1 min at 600 rpm in a microplate shaker (Eppendorf, Hamburg, Germany) and incubated for 30 min in the dark at room temperature. After incubation, the absorbance was measured at 517 nm on the microplate reader. The scavenging activity was calculated using the following Equation (2):

(2) $$\%\;SA=\left(1-\left(\frac{A\;\left(sample\right)-(A\left(blank\right)}{A\left(neg.control\right)}\right)\right)\times 100$$

### 4.11. Statistical Analysis

All the experiments were carried out in triplicate as three or more independent experiments. The data are expressed as means ± standard deviation, with analysis using Origin 2018 (OriginLab, Northampton, MA, USA). Statistical analysis was performed using IBM SPSS Statistics 23 (Statsoft Inc., Tulsa, OK, USA). In order to determine the distribution and homogeneity of the data, the Kolmogorov–Smirnov test of normality and the homogeneity of variances test were performed. The data were normally distributed and variances were equal across groups. Statistical significances were determined using the One-Way ANOVA test. Data were accepted as significant at *p* < 0.05. Pearson’s correlation test was used to determine the correlation between MIC and *C. jejuni* intercellular signaling, adhesion, and biofilm formation. The correlation was significant at the level *p* < 0.01.

## 5. Conclusions

This comparative study aimed to find antimicrobials that were potentially able to reduce *C. jejuni* biofilm establishment on abiotic surfaces. Prepared EOs, EFs, and EWMs of *Lavandula × intermedia* ‘Bila’, ‘Budrovka’ SN, and ‘Budrovka’ showed great antibacterial activity against one of the major food-borne pathogens, *C. jejuni*. Their effect against *C. jejuni* intercellular signaling, adhesion, and biofilm formation at subinhibitory concentration was confirmed, making lavandin formulations antimicrobial agents that can be used as an innovative approach to control *C. jejuni* biofilm development. Moreover, a correlation was confirmed between the reduction of *C. jejuni* intercellular signaling and the reduction of *C. jejuni* adhesion, two interrelated properties that can be easily controlled simultaneously. It is important to emphasize that lavandin ethanolic extracts showed better activity against *C. jejuni* intercellular signaling and adhesion, as well as better antioxidant activity, which makes them competitive with EOs. These findings mean that novel bacterial targets are of interest for biofilm control with alternative natural agents throughout the whole food production chain.

## Figures and Tables

**Table 1:** Identification of the main common phenolic compounds in the lavandin ethanolic extract (EF) and lavandin (EWM).

No.	Rt	Compound Identified	Full Scan MS (*m/z*)	Fragment Ions (MS^2^; *m/z*)	UV Maximum (nm)
1	4.97	3-(3,4-Dihydroxyphenyl)lactic acid *	395 [M+HCOOH-H]^−^, 197 [M-H]^−^	197 (100)	225 sh, 281
2	8.34	Coumaric acid hexoside I	325 [M-H]^−^	163 (100), 119 (25)	263, 290 sh
3	10.23	Ferulic acid hexoside I	355 [M-H]^−^	193 (100), 149 (20)	302
4	10.23	Caffeic acid hexoside	387 [M+HCOOH-H]^−^	341 (100), 207 (25)	302
5	12.13	Coumaric acid hexoside II	371 [M+HCOOH-H]^-^, 325 [M-H]^−^	325 (100)	277, 290 sh
6	14.21	Ferulic acid hexoside II	401 [M+HCOOH-H]^−^, 355 [M-H]^−^	355 (100)	295, 319
7	18.87	Apigenin-7-O-glucoside	431 [M-H]^−^	269 (100)	268, 334
8	19.60	Rosmarinic acid	359 [M-H]^−^	161 (100), 179 (30), 223 (10)	292 sh, 328
9	23.96	Salvianolic acid A *	493 [M-H]^−^	295 (100), 313 (10)	287, 340 sh
10	30.24	Ladanein (5,6-di-OH-7,4’-dimethoxy flavone)	315 [M-H]^−^	300 (100)	284, 333

* Not detected in lavandin ethanolic extract (EF).

**Table 2:** Identification of the main components of the lavandin EOs.

			Quantification of Total ^c^		
Retention Time	Retention Index ^a^	Compound ^b^	*Lavandula × Intermedia* ‘Bila’	*Lavandula × Intermedia* ‘Budrovka’ SN	*Lavandula × Intermedia* ‘Budrovka’
5.126	926	α-Thujene	0.086	0.139	0.012
5.301	932	α-Pinene	0.635	0.813	0.317
5.680	946	Camphene	0.248	0.33	0.031
6.349	973	Sabinene	0.173	0.196	0.178
6.442	975	β-Pinene	0.882	0.98	0.685
6.710	979	3-Octanon	tr	tr	0.601
6.851	991	Myrcen	0.27	0.311	0.343
7.449	1010	Δ3-Carene	0.235	0.222	tr
7.925	1023	p-Cymene	0.275	0.263	0.132
8.126	1030	1,8-Cineol	14.091	12.585	14.211
8.378	1037	(Z)-β-Ocimene	2.565	4.047	0.44
8.754	1047	(E)-β-Ocimene	0.128	0.27	0.046
9.115	1058	γ-Terpinene	0.104	0.165	tr
9.392	1065	cis-Sabinenhydrate	0.059	0.088	0.025
9.595	1071	cis-Linalool oxide (furanoid)	tr	0.027	0.116
10.187	1087	trans-Linalool oxide (furanoid)	tr	tr	0.097
10.198	1088	Terpinolene *	0.14	0.22	n.d.
10.662	1100	Linalool	40.412	43.058	47.206
11.141	1112	Octen-3-yl-1-acetate	0.05	0.024	0.235
12.323	1142	Camphor	2.998	1.371	0.383
12.616	1150	Hexyl-2-methylpropanoate	0.099	0.087	0.135
13.209	1163	endo-Borneol + Lavandulol	13.946	14.053	0.121
13.706	1175	Terpinen-4-ol	8.37	8.813	0.962
14.271	1190	α-Terpineol	1.159	0.827	1.106
14.418	1192	Hexylbutanoate	0.753	0.739	0.628
15.793	1227	endo-Bornylformiate *	0.23	0.171	n.d.
16.290	1237	Hexyl-2-methylbutanoate	0.206	0.194	0.276
16.497	1242	Hexylisovalerate	0.058	0.047	0.128
17.069	1256	Linalylacetate	6.645	5.264	26.709
18.577	1291	Lavandulylacetate	0.879	0.693	0.105
22.607/22.676	1387/1390	Hexylhexanoate + 7-epi-Sesquiphellandrene	0.148	0.118	0.069
23.773	1419	trans-Caryophyllene	0.663	0.625	1.704
25.423	1457	(E)-β-Farnesene	2.611	2.151	0.123
25.782	1466	Lavandulyl-butanoate *	0.102	0.051	n.d.
26.265	1480	Germacrene D	0.357	0.431	0.162
27.549	1510	Lavandulyl-isovalerate *	0.309	0.219	n.d.
30.174	1579	trans-Caryophyllenoxide	tr	0.018	0.341
34.016	1682	α-Bisabolol	tr	0.033	0.89

^a^ Linear Retention Index relative to n-alkanes on HP-5-MS column; ^b^ Compounds identified by mass spectral libraries [23,24]; ^c^ Quantification by normalization (area percent method) without considering calibration factors. tr—in traces; n.d.—not determined; * not determined in *Lavandula × Intermedia* ‘Budrovka’.

**Table 3:** Minimal inhibitory and subinhibitory concentration determined against *C. jejuni* NCTC 11168 and *C. jejuni* 11168ΔluxS for the lavandin formulations (EOs, EFs and EWMs).

	*C. jejuni* NCTC 11168		*C. jejuni* 11168ΔluxS	
Sample	MIC (mg/mL)	0.25 × MIC (mg/mL)	MIC (mg/mL)	0.25 × MIC (mg/mL)
EO ‘Bila’	0.25	0.062	0.25	0.062
EO ‘Budrovka’ SN	0.25	0.062	0.25	0.062
EO ‘Budrovka’	0.25	0.062	0.25	0.062
EF ‘Bila’	0.5	0.125	0.25	0.062
EF ‘Budrovka’ SN	1	0.25	0.5	0.125
EF ‘Budrovka’	0.5	0.125	0.5	0.125
EWM ‘Bila’	1	0.25	1	0.25
EWM ‘Budrovka’ SN	1	0.25	1	0.25
EWM ‘Budrovka’	1	0.25	1	0.25

## Data Availability

The data is available in supplementary materials.

## Supplementary Materials

The following supporting information can be downloaded at https://www.mdpi.com/article/10.3390/antibiotics11070854/s1: Method S1. LC-MS Conditions; Method S2. GC–MS Conditions; Table S1. Growth of *C. jejuni* NCTC 11168 and *C. jejuni* 11168ΔluxS in MH broth without or with the addition of lavandin formulations (EOs, EFs and EWMs) at subinhibitory concentration (0.25 × MIC) after 24 h of incubation in a micro-aerobic atmosphere. Average values of CFU/mL are shown ± SD; Table S2. Reduction of *C. jejuni* NCTC 11168 intercellular signaling after the addition of lavandin formulations (EOs, EFs and EWMs) at subinhibitory concentration (0.25 × MIC). Average values in % are shown ± SD; Figure S1. Yield of ethanol extraction for lavandin ethanolic extracts, i.e., lavandin EFs and lavandin EWMs. Figure S2. HPLC chromatogram (UV 310 nm) of the ethanolic extract of lavandin flowers prior to distillation [EFs]. * not identified (no significant ionization in ESI positive and negative mode); compound 1 was not detected; for identity of compounds 2–10, please refer to Table 1. Figure S3. HPLC chromatogram (UV 320 nm) of the ethanolic extract of lavandin postdistillation waste materials (EWMs). * not identified (no significant ionization in ESI positive and negative mode); compound 1 (3-(3,4-OH-phenyl)lactic acid was not detectable at 310 nm, but was detected in the ESI-MS base peak chromatogram; for identity of compounds 2–10, please refer to Table 1. Figure S4. Comparison of peak areas of compounds 2–10 in EF and EWM samples. Peak areas were calculated from UV chromatograms at 310 nm.
