# Supplementary material for: Campylobacter jejuni Biofilm Control with Lavandin Essential Oils and By-Products
Source: Antibiotics (Basel). 2022 Jun 25;11(7):854. doi: 10.3390/antibiotics11070854 (PMC9312237; doi:10.3390/antibiotics11070854)
Supplement: Supplementary file 1 [file antibiotics-11-00854-s001.zip › antibiotics-1782413-supplementary.pdf]

Supplementary Information

Supplementary Methods

Method S1. LC-MS Conditions

The dry ethanolic extracts from the flowers and the waste material (5 mg) were dissolved in 1ml ethanol and centrifuged prior to analysis. This analysis was performed on an HPLC system (Ultimate RS 3000 Dionex) coupled to a mass spectrometer (LTQ XL; Thermo Scientific, Waltham, USA). The column (Luna phenyl-hexyl; 5 µm; 250 × 2 mm; Phenomenex, Torrance, CA, USA) was used with a gradient elution of 0.1% formic acid in water (eluent A) and acetonitrile (eluent B), as: 0→20 min, 10%→70% B; 20→40 min, 70%→100% B; 40→45 min, 100% B; 45→45.5 min, 100%→10% B; and 45.5→52 min, 10% B. The flow rate was 0.250ml/min and the column was maintained at 35°C. Mass spectra were recorded in negative ion mode for the m/z range from 50 to 2000 amu, with data-dependent fragmentation (normalized collision energy, 35%). Mass spectrometry conditions were set to: capillary temperature, 350°C; source temperature, 300°C; sheath and auxiliary gas flow, 40 and 10 arbitrary units (machine settings) respectively; source voltage, 3.5 kV; and capillary voltage, -17 V.

Method S2. Gas Chromatography–Mass Spectrometry (GC-MS) Conditions

Essential oil (10 µL) was dissolved in 990 µL hexane and further analysed by gas chromatography (7890 A; Agilent Technologies, Santa Clara, USA) and mass spectrometry (5975 C VL MSD; Agilent Technologies, USA), operating at 70 eV, with ion source temperature 230°C, and interface temperature 280°C. A split injection (injection volume, 0.2 µL; split ratio, 50:1) at 240°C injector temperature was used for essential oils. A fused silica capillary column was used (5% phenyl, 95% methyl polysiloxane; HP-5MS; 30 m × 250 µm × 0.25 µm; Agilent J & W, USA). The temperature programme was as follows: 1 min at 60°C, then raised to 220°C at 3°C/min. The carrier gas was helium 5.6 at a flow rate of 0.9ml/min. Data acquisition was performed using Agilent GC/MSD ChemStation Version E.02.02 for the mass scan range of 40 u to 400 u.

Supplementary Tables and Figures

Table S1. Growth of *C. jejuni* National Collection of Type Culture (NCTC) 11168 and *C. jejuni* 11168Δ*luxS* in MH broth without or with the addition of lavandin formulations (essential oils [EOs], ethanolic extracts of lavandin flowers prior to distillation [EFs] and ethanolic extracts of lavandin post-distillation waste material [EWMs]) at subinhibitory concentration (0.25 × MIC) after 24 hours of incubation in a micro-aerobic atmosphere. Average values of CFU/ml are shown ±SD.

**Sample**	**Average CFU/ml**	**Standard deviation**
	C. jejuni NCTC 11168	
C. jejuni NCTC 11168	1,55E+08	7,82E+07
EO 'Bila'	2,23E+08	2,60E+07
EO 'Budrovka' SN	1,91E+08	6,43E+07
EO 'Budrovka'	1,21E+08	2,28E+07
EF 'Bila'	8,44E+07	1,29E+07
EF 'Budrovka' SN	1,48E+08	2,04E+07
EF 'Budrovka'	5,72E+07	3,40E+07
EWM 'Bila'	1,56E+08	8,53E+07
EWM 'Budrovka' SN	1,09E+08	2,11E+07
EWM 'Budrovka'	1,07E+08	6,59E+07
	C. jejuni 11168Δ*luxS*	
C. jejuni 11168Δ*luxS*	1,57E+08	4,66E+07
EO 'Bila'	8,22E+07	3,14E+06
EO 'Budrovka' SN	1,22E+08	3,71E+07
EO 'Budrovka'	1,31E+08	7,86E+06
EF 'Bila'	1,38E+08	8,75E+06
EF 'Budrovka' SN	7,78E+07	1,77E+07
EF 'Budrovka'	1,58E+08	8,31E+06
EWM 'Bila'	1,91E+08	1,93E+07
EWM 'Budrovka' SN	1,13E+08	2,76E+07
EWM 'Budrovka'	1,89E+08	1,85E+07

Table S2. Reduction of *C. jejuni* NCTC 11168 intercellular signalling after the addition of lavandin formulations (essential oils EOs], ethanolic extracts of lavandin flowers prior to distillation [EFs] and ethanolic extracts of lavandin post-distillation waste material [EWMs]) at subinhibitory concentration (0.25 × MIC). Average values in % are shown ±SD.

**Sample**	**Reduction of *C. jejuni* intercell. signalling** [%]
EO 'Bila'	58.23 ± 5.71
EO 'Budrovka' St Nicholas (SN)	60.37 ± 8.18
EO 'Budrovka'	59.58 ± 2.18
EF 'Bila'	94.01 ±8.76
EF 'Budrovka' SN	88.27 ± 16.76
EF 'Budrovka'	95.26 ± 12.64
EWM 'Bila'	94.33 ± 14.93
EWM 'Budrovka' SN	68.34 ± 4.56
EWM 'Budrovka'	58.97 ± 3.54

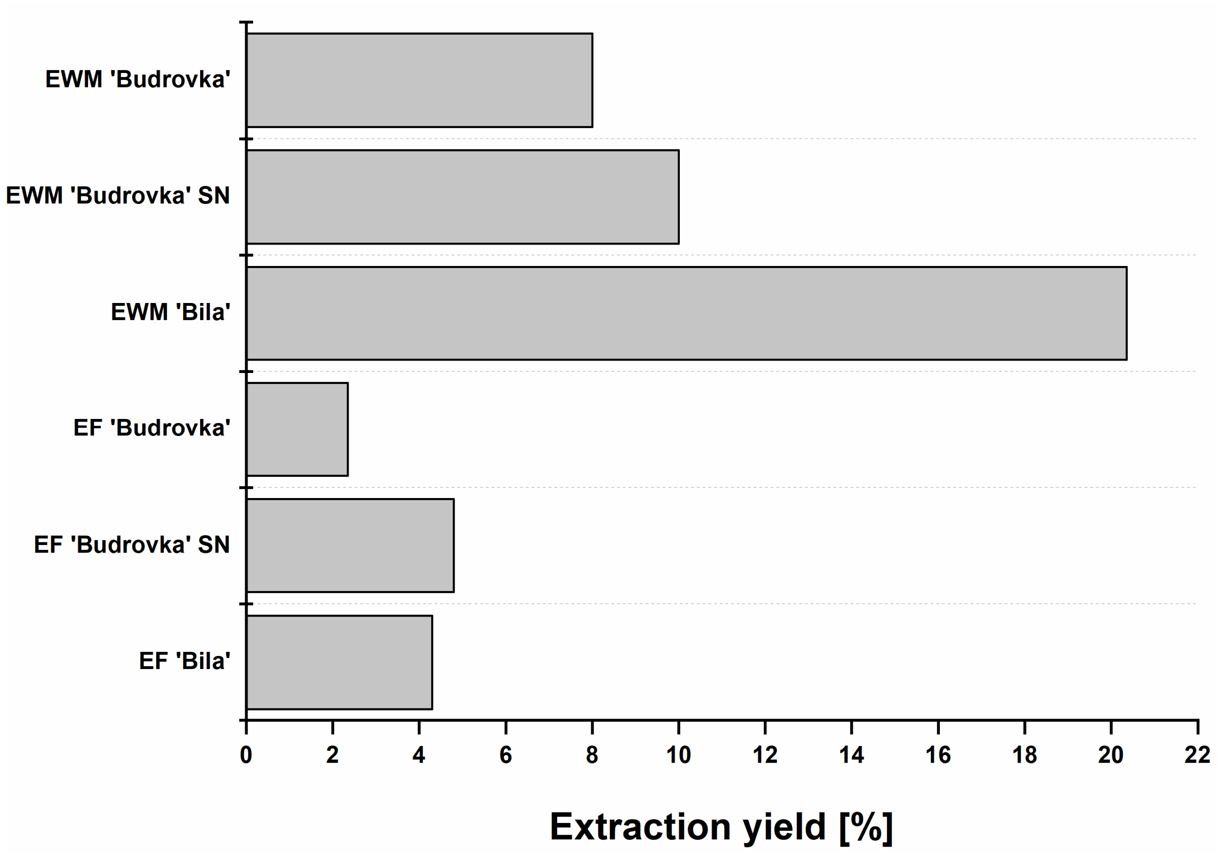

Figure S1. Yield of ethanol extraction for lavandin ethanolic extracts, i.e. lavandin ethanolic extracts of flowers prior to distillation (EFs) and lavandin ethanolic extracts of post-distillation waste material (EWMs)

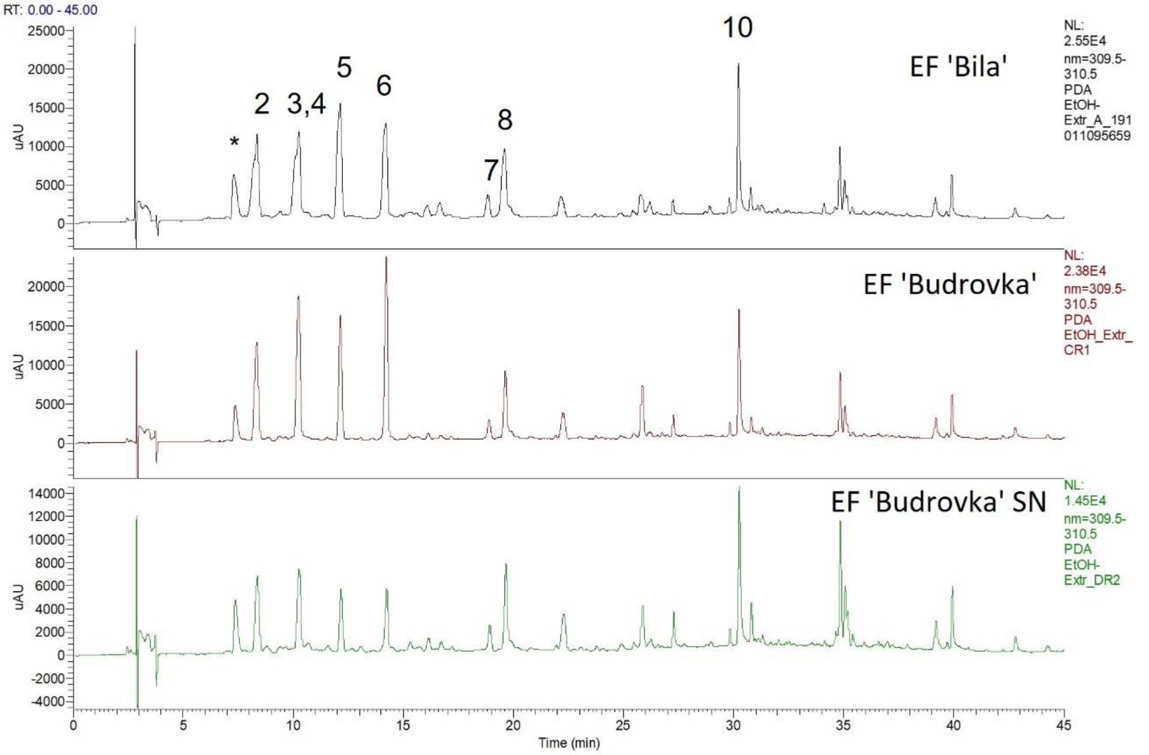

Figure S2. UHPLC-UV (310 nm) chromatograms of EF samples. * not identified (no significant ionization in ESI positive and negative mode); compound **1** could not be detected; for identity of compounds **2** -**10**, please refer to Table 1.

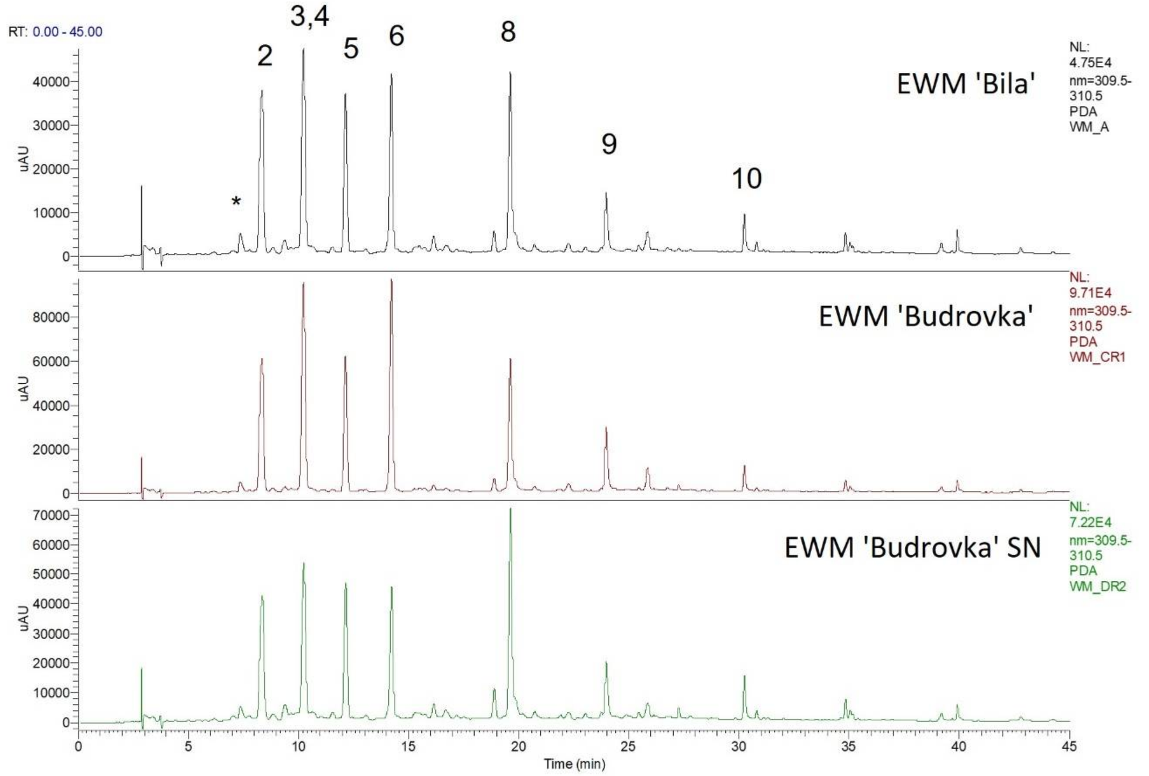

Figure S3. UHPLC-UV (310 nm) chromatograms of EWM samples. * not identified (no significant ionization in ESI positive and negative mode); compound **1** (3-(3,4-OH-phenyl)lactic acid was not detectable at 310 nm, but was detected in the ESI-MS base peak chromatogram. For identity of compounds **2 -10**, please refer to Table 1.

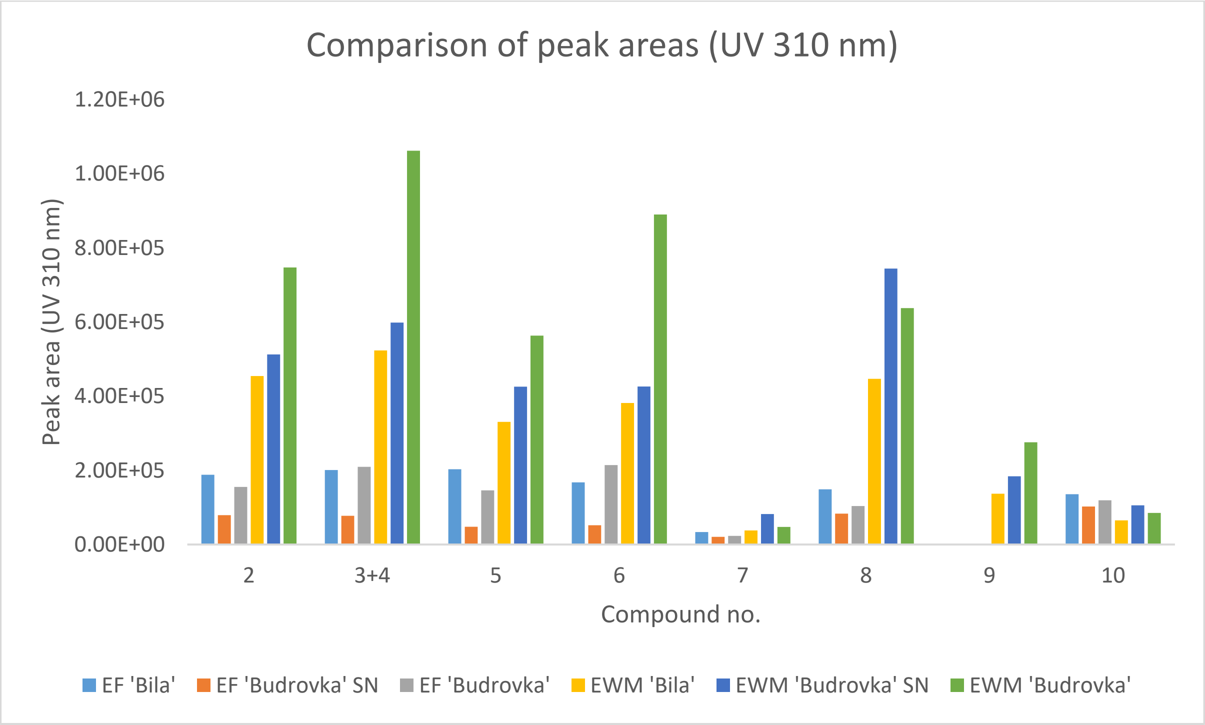

Figure S4. Comparison of peak areas of compounds **2** – **10** in EF and EWM samples. Peak areas were calculated from UV chromatograms at 310 nm.
